# ^18^F-FDG PET/CT as a molecular biomarker in the diagnosis of amyotrophic lateral sclerosis associated with prostate cancer and progressive supranuclear palsy: A case report

**DOI:** 10.3389/fnume.2023.1137875

**Published:** 2023-04-17

**Authors:** Emilly A. Cortés Mancera, Fabio A. Sinisterra Solis, Francisco R. Romero-Castellanos, Ivan E. Diaz-Meneses, Nora E. Kerik-Rotenberg

**Affiliations:** PET/CT Molecular Imaging Unit. National Institute of Neurology and Neurosurgery, Mexico City, Mexico

**Keywords:** PET/CT, amyotrophic lateral sclerosis, FDG, prostate cancer, PSMA, palsy, supranuclear

## Abstract

**Introduction:**

Amyotrophic lateral sclerosis (ALS) is a neurodegenerative, multisystem disorder. Its clinical presentation typically consists of progressive focal muscle atrophy and weakness. In addition to motor disorders, the association between ALS and cancer has been researched, such as frontotemporal dementia and progressive supranuclear palsy. The diagnosis is based primarily on the clinical history, physical examination, electrodiagnostic tests (with an EMG needle), and neuroimaging, such as MRI and ^18^F-FDG PET/CT.

**Presentation of the case:**

A 67-year-old male patient was diagnosed with prostate adenocarcinoma with a clinical picture of muscle weakness in the lower limbs that caused falls and was associated with fasciculations in the thighs and arms, alterations in the tone of voice, poor memory, and difficulty articulating words. In the neurological assessment, he described walking supported by a walker with decreased strength in both lower limbs and sensitivity without alterations. The diagnoses of upper and lower motor neuron disease and probable ALS were integrated. Furthermore, the probable coexistence of frontotemporal dementia/disorder (FDD) with ALS was considered. The main findings in the ^18^F-FDG PET/CT study was hypometabolism in the cortex of the bilateral motor and premotor areas, the anterior cingulate, both caudate and putamen, a metabolic pattern compatible with ALS, and progressive supranuclear palsy.

**Conclusion:**

Through the PET/CT studies, we demonstrated a case in which ALS, prostate cancer and progressive supranuclear palsy coexisted molecularly; it was clinically difficult to diagnose. Molecular imaging has potential in the diagnostic and prognostic evaluation of ALS. It is crucial to identify the disease early and reliably through metabolic patterns that allow us to confirm the disease or differentiate it from other pathologies.

## Introduction

Amyotrophic lateral sclerosis (ALS) is considered a fatal disease. Over time, the term “amyotrophic lateral sclerosis” has undergone various modifications in its definition, which since 1869 has been considered to be pure motor neuron disease (1). However, it is currently known as a progressive multisystem neurodegeneration leading to the death of motor neurons, characterized by significant muscle weakness and a poor prognosis.

The clinical manifestations are highly variable, but they generally begin in the extremities with upper and lower motor neuron signs (2). The average age of onset is in the range of 57–62 years. Muscle weakness starts mainly in the distal muscles rather than in the proximal muscles. Some patients may develop bulbar onset of the disease with dysarthria and dysphagia, and a smaller percentage may only present with isolated primary lateral sclerosis and progressive muscular atrophy (3). The disease is progressive and fatal in most patients. Once symptoms start and a diagnosis is confirmed, approximately 50% of patients with ALS have a survival rate of 3 years or more after diagnosis (4), approximately 25% live 5 years or more, and up to 10% live more than 10 years; mortality is due in most cases to respiratory muscle failure. In addition to this, extra-motor manifestations have recently been studied more frequently, the percentage of which is increasing and reaching up to 50% of patients. At the neurological level, ALS has been related to various pathologies, the most frequent being frontotemporal dementia (FTD), present in 10–15% of cases and up to 40% can present with cognitive, behavioral and executive alterations (5), cases of primary progressive aphasia and progressive supranuclear palsy have been documented (6–8).

On the other hand, some studies refer to the relationship between ALS neurodegeneration and carcinogenesis. Regarding the pathophysiology of cancer, there is a loss in the regulation of apoptosis, which is why cancer cells lead to resistance to death, generating uncontrollable and disorganized growth, while the neurodegeneration caused by ALS is linked to irreversible cell death. Within neurodegenerative diseases, Parkinson's disease (PD) and Alzheimer's disease (AD) are the most studied in terms of their inverse relationship with carcinogenesis, but little has been studied about ALS, even though it is a rare disease that has generated interest in how it relates to various types of cancer (9, 10).

The phenotypic, clinical, molecular, and genetic heterogeneity of ALS makes an early diagnosis difficult. Despite this, the diagnosis is based primarily on the clinical history, neurological examination, imaging studies such as magnetic resonance imaging (MRI), serological tests, and electrodiagnosis (needle electromyography (EMG)), the latter to confirm the involvement of the lower motor neurons (11, 12).

According to the Food and Drug Administration-National Institutes of Health (FDA-NIH) Biomarkers Working Group, a biomarker is a measure of the biochemical, functional, biological, cellular, and molecular processes of the disease state (13, 14). Positron emission tomography/computed tomography (PET/CT) is not considered part of the diagnostic criteria; however, it is having an increasing impact on molecular diagnosis through its radiopharmaceuticals, with ^18^F-FDG being the most widely used to assess brain glycolytic metabolism, which is considered a molecular biomarker for ALS (15).

In recent years, various studies have demonstrated the diagnostic potential of ^18^F-FDG PET in the diagnosis and prognosis of patients with ALS (16–18).

Worldwide, prostate cancer is the second most common cancer and is considered the sixth leading cause of cancer-related death. According to the most recent GLOBOCAN report in 2020, approximately 1,414,259 (7.3%) new cases were registered. Timely diagnosis has increased thanks to the detection of prostate-specific antigen (PSA) (19). The use of molecular images in patients with prostate cancer has been fundamental in the diagnosis, follow-up, prognosis and treatment of patients, especially with radiotracers that bind to prostate-specific membrane antigen such as ^18^F-PSMA-1007 or ^68^Ga-PSMA, which have been recommended by different guidelines as an adequate method to evaluate prostate, lymph node and bone involvement in order to provide the greatest therapeutic benefit, including therapy with ^177^Lu-PSMA, which in recent years has shown an increase in the overall survival (20, 21).

## Case report

A 67-year-old unemployed, Catholic, right-handed man with a 7-year history of primary hypothyroidism treated with levothyroxine 75 µg and intermediate-risk prostate adenocarcinoma (Gleason 4 + 3 = 7 and ISUP 3), with a low prostate-specific antigen (PSA) of 0.01 ng/dl, rising to 2 ng/dl, and last reported at 4.5 ng/dl with a doubling time of 3 months, was treated with radical prostatectomy and subsequent hormonal blockade (6-monthly application of leuprorelin). A clinical picture of 8 months of evolution is added with muscle weakness and fasciculations in the lower limbs that led to falls, 1 month after these symptoms he presented changes in the tone of voice and difficulty articulating words..

In addition, the patient initially presented dysphagia with liquids, which has progressed to solids. In the last month, he has experienced increased difficulty in breathing and in fasciculations. He has noticed weight loss, with a decrease in muscle mass very noticeable in his legs. In the neurological assessment, he was described as follows: alert; oriented in person, time, place, and circumstance; not fluent in the language, names, repetition, and understanding; flaccid dysarthria; judgment and abstraction without alliteration; and unaltered semantic and declarative memory. His gait was supported by a walker. In the evaluation of the cranial nerves, ocular movements with a limitation to supraversion, hypometric saccades, increased masseteric reflex, a central uvula, asymmetric elevation of the soft palate, decreased gag reflex, a tongue with hypotrophic edges, and fasciculations at rest were found. The strength in the upper extremities was preserved but the strength in the lower extremities was decreased by 3/5 proximal and 4/5 distal. He had an unaltered tone. Muscle tone without alterations. Hypotrophy in interosseous muscles and pelvic limbs. Biceps, triceps, styroradial, patellar, and bilateral Achilles reflexes were increased (+++/++++). There were fasciculations in all four extremities and in the tongue. Exteroceptive and proprioceptive sensation without alterations. Therefore, they integrated the following diagnoses 1. upper motor neuron syndrome and 2. lower motor neuron syndrome, probable ALS.

The neuropsychiatric assessment documented easy crying, sometimes feeling unmotivated, sometimes accompanied by laughter, and a greater deterioration of speech and memory (the patient writes on a cell phone to communicate). In addition to the probable picture of ALS, the probable coexistence of FTD was also considered.

**Figure 1:**
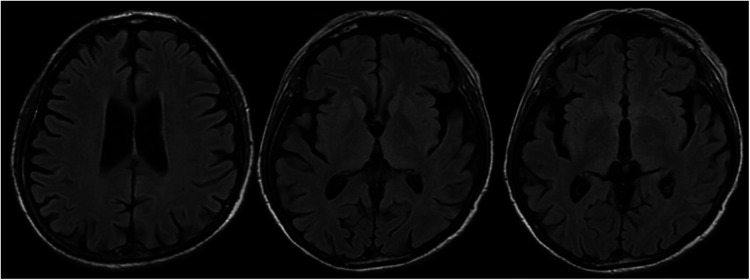
Brain FLAIR MRI. Axial section showing global cortical atrophy, mainly in the right temporal lobe.

**Figure 2:**
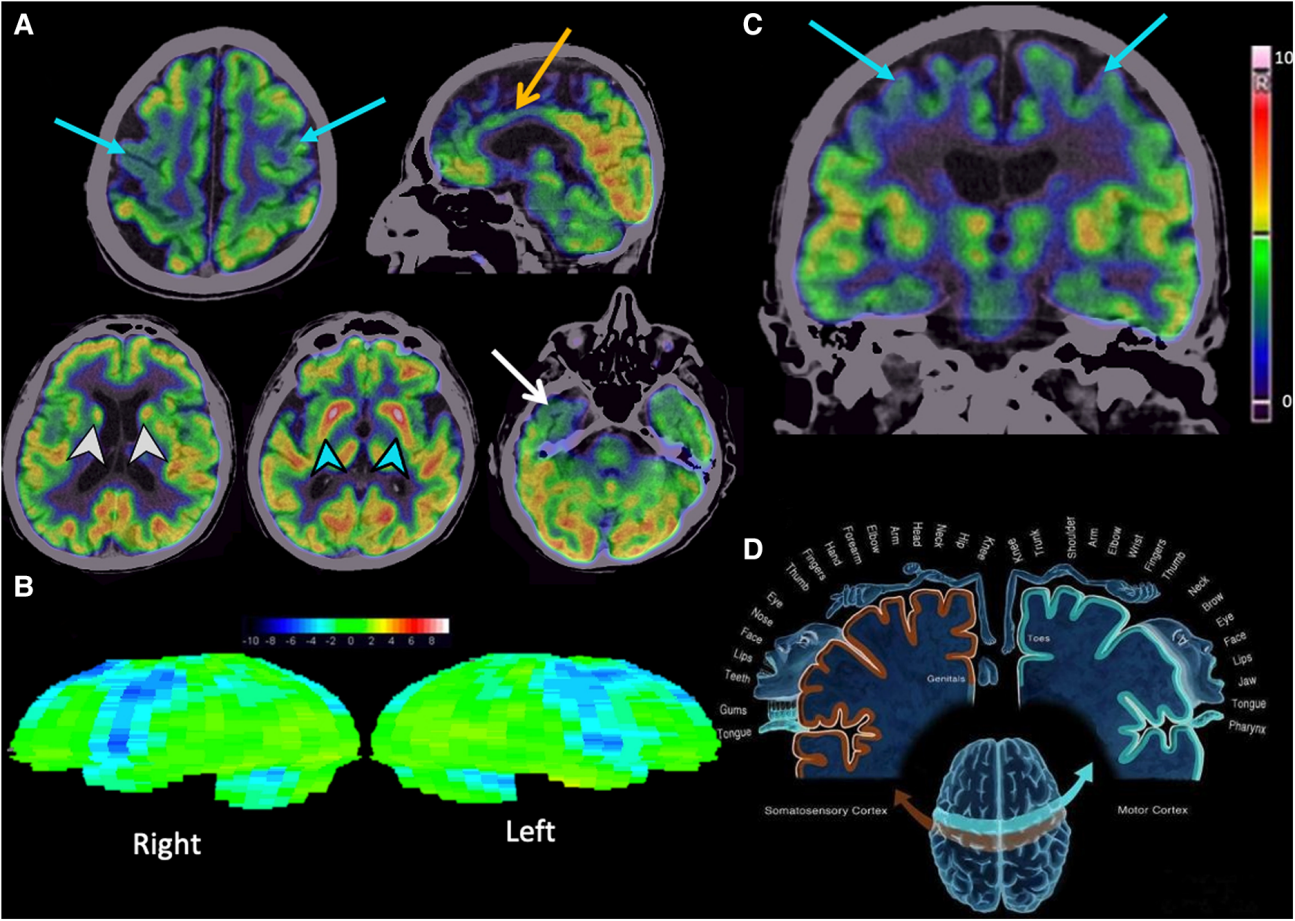
Brain 18F-FGD PET/CT. (**A**) Axial and sagittal sections showing moderate to severe hypometabolism in the cortex of the primary motor area and bilateral premotor area (blue arrows), moderate hypometabolism in the bilateral anterior cingulate (yellow arrow), and mild to moderate hypometabolism in the right temporal neocortex in its polar portion with extension to the entorhinal cortex (white arrow). Basal nuclei with mild to moderate hypometabolism in the heads of both caudates (white arrowheads) and moderate hypometabolism of both putamen in their middle and posterior thirds (blue arrowheads). (**B**) Syngo Scenium corroborates hypometabolism in the bilateral motor and premotor cortex. (**C,D**) Coronal section showing hypometabolism in the primary motor cortex correlating with the cortical or Penfield homunculus.

**Figure 3:**
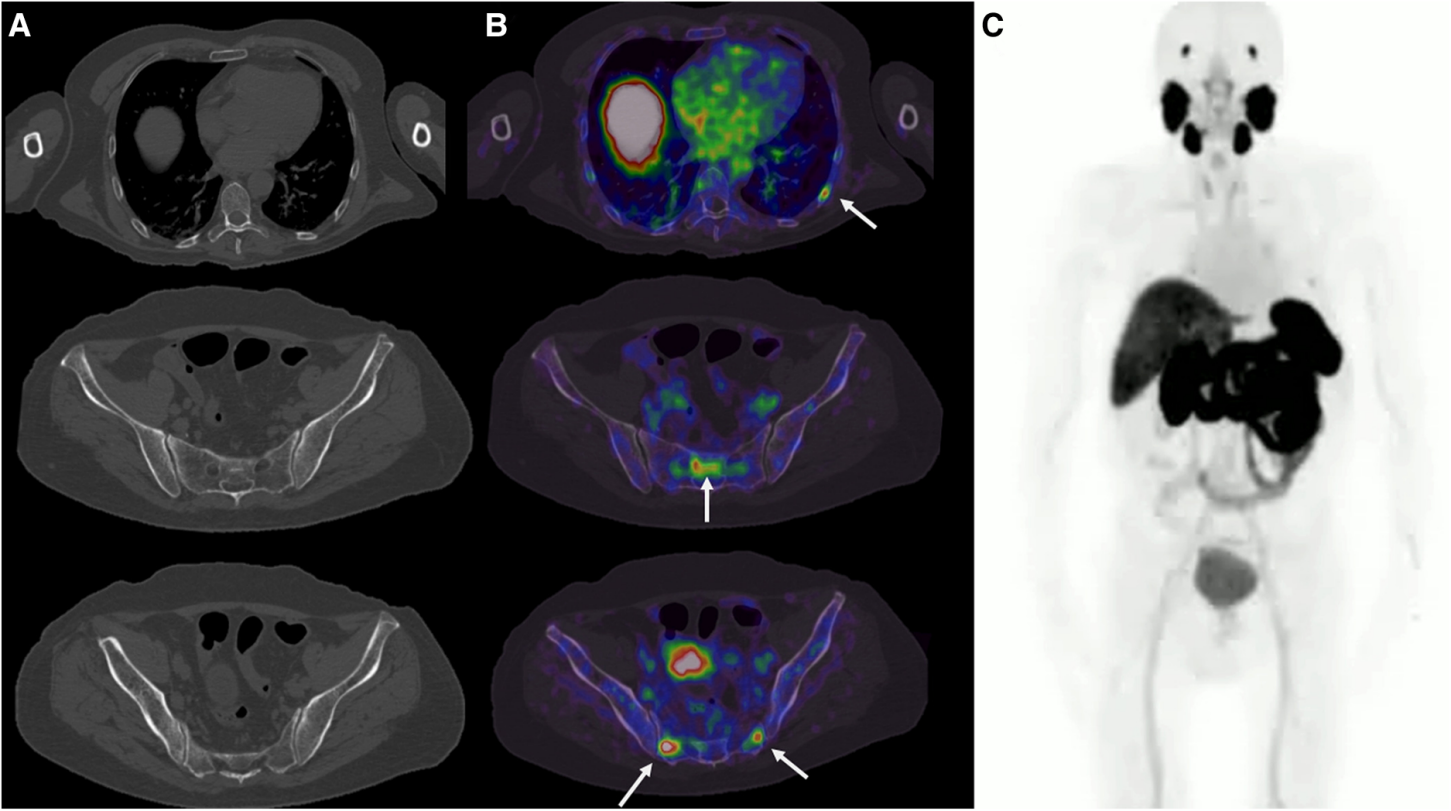
18F-PSMA-1007 PET/CT. (**A**) Axial slices of plain tomography without blastic bone lesions. (**B**) PET/CT shows focal areas of radiotracer uptake in the left posterior seventh costal arch and sacrum, without morphologic translation. (**C**) Maximum intensity projection with 18F-PSMA-1007.

It complements the diagnostic approach with EMG, which showed fibrillations and positive sharp waves, fasciculation potentials, increased amplitude and polyphasia. The MRI scan showed only data of generalized atrophy (Figure 1) and the ^18^F-FDG PET/CT scan of the brain (Figure 2) showed decreased metabolism in the cortex of the primary motor area and bilateral premotor, bilateral anterior cingulate, mild to moderate right temporal neocortex in its polar portion with extension to the entorhinal cortex, both caudates, and the posterior third of the putamen. Due to the antecedent of prostatic adenocarcinoma and suspicion of biochemical recurrence, an 18F-PSMA-1007 PET/CT scan was performed, which showed at least four bone-level uptake sites in the left seventh costal arch and sacrum without morphological translation (Figure 3).

## Discussion

Generally, ALS poses multiple challenges in its diagnosis, treatment, and prognosis, with an unfavorable neurodegenerative impact on the quality of life of patients, mainly affecting the motor system, although non-motor manifestations have been increasingly documented. Destruction of the upper and lower motor neurons in the motor cortex, brainstem nuclei, and spinal cord results in atrophy and progressive muscle weakness for which treatment is still very unsatisfactory, hence the poor prognosis. Recently, questions have been raised about the link between cancer and neurodegeneration, in the case of ALS, which has a genetic cause. There is evidence for the involvement of several oncogenes, for example, SQSTM1 (22), UBQLN2 (23), FUS (24), and GRN (25).

In a study carried out by Gibson et al., they included 1,081 cases of ALS, of which they observed 114 (10.5%) cases with a diagnosis of independent primary cancer. They determined that lung cancer had the lowest risk (hazard ratio (HR) 0.23, *p* = 0.002, 95% confidence interval (CI) 0.05–0.63) and salivary gland cancer (HR 5.27, *p* = 0.041, 95% CI 1.09–15) and testicular cancer (HR 3.82, *p* = 0.042, 95% CI 1.06–9.62) (26) had a relatively high risk.

In our patient, the diagnosis of prostate cancer and the onset of motor symptoms were practically simultaneous. 18F-PSMA PET/CT showed the focal areas at the bone level with an increased uptake of the radiotracer (maximum standardized uptake value 14.3–20.3) (Figure 3), which did not have a morphological translation. Unfortunately, further imaging was not performed, so these bone lesions could not be confirmed; however, given the high PSA of 4.5 ng/dl and the nadir of 0.01 ng/dl, with a doubling time of 3 months, it was considered a biochemical recurrence. Therefore, the findings described with 18F-PSMA-1007 PET/CT are highly suggestive of metastatic prostate cancer. Depending on the time of the diagnosis of prostate cancer, there is an inversely proportional relationship with death from ALS with low risk, unlike others such as tongue cancer and melanoma (10). However, in a more recent study, the risk of ALS was generally not associated with the individual cancer sites examined, including prostate cancer (27). The presence of insulin-like growth factor I (IGF-1) is considered a risk factor for the development and growth of prostate cancer (28) due to its signaling through RAS/RAF/MAPK (29); however, due to its neurotrophic role, it is essential for normal development and plasticity in the nervous system, which is why it has been the subject of investigation for its possible therapeutic and protective effects on ALS (30). Most studies support a potential protective and therapeutic effect in ALS with improved survival (31–33). Unfortunately, in a clinical trial of 330 patients in which IGF-1 was administered subcutaneously, the effects of IGF-1 on muscle strength and survival were observed, and IGF-1-treated patients did not improve in relation to the controls (34).

To make the diagnosis of ALS, the predominant clinical findings involving the upper and lower motor neurons must be integrated, causing hyperreflexia, weakness, and muscle atrophy, respectively, with the presence of non-motor symptoms, as is the case with our patient with motor and extra-motor manifestations, such as muscle weakness and language disturbances, in addition to the biochemical data on the recurrence of prostate cancer with bone metastases. For many years, molecular imaging has been the mainstay in the field of oncology; however, it currently stands out widely in the neurological field. In 1987, the first PET/CT study with ^18^F-FDG was performed, and the metabolic findings of patients with ALS and the involvement of the upper motor neurons were described, showing decreased metabolism in the primary motor cortex compared to healthy patients (35). This finding is not the only one; other metabolic alterations have been described, such as hypometabolism in the supplementary motor cortex and the premotor cortex, and the extension to the parietal and frontal lobes (36, 16).

^18^F-FDG PET-CT findings of the patient (Figure 2) revealed a typical pattern described in ALS (bilaterally hypometabolism in the primary motor cortex and premotor cortex), which we could call the “hypometabolic band sign”. In addition, other findings were found that have not been described in ALS (hypometabolism in the anterior” cingulate and striatum). These findings have been described in progressive supranuclear palsy (PSP), for which we consider the coexistence of these two neurodegenerative entities. Therefore, ^18^F-FDG PET/CT has the advantage over other imaging modalities in diagnosing the ALS pattern with other co-morbidities, such as this case of PSP.

According to a multicenter study conducted by Van Weehaeghe et al. that included 105 patients diagnosed with ALS, they found that ^18^F-FDG PET/CT has a sensitivity of 100%, a specificity of 95%, and an accuracy of 95% for the diagnosis of ALS (15).

For many years, PET/CT has been considered a molecular biomarker due to the use of its radiotracers, whose objective is to visualize molecular processes *in vivo*, therefore, at the neurological level we can evaluate synaptic and molecular behavior. For this reason, various scientific publications have taken on the task of investigating its role in ALS. One of the largest cohorts of 195 patients to date compared healthy controls and patients with ALS, highlighting its high sensitivity of 95.4% and specificity of 82.5% (16); therefore, unlike other imaging modalities, ^18^F-FDG PET/CT allows the early evaluation of metabolic changes at the brain level and the integration of patterns that suggest the coexistence of other diseases. MR studies have also considered it to be a promising biomarker for ALS, especially diffusion tensor imaging (DTI); however, a meta-analysis (37) concludes that it has a low sensitivity of 65% and a specificity of 67%.

## Conclusion

Here we demonstrated a different case in which ALS and PSP coexisted clinically and metabolically according to the findings of ^18^F-FDG PET/CT. A comprehensive assessment was made concurrently with the performance of 18F-PSMA-1007 using the history of prostate adenocarcinoma with bone metastases. Because molecular imaging has shed light on the pathophysiology of ALS, it has the potential to fill a critical gap in the diagnostic and prognostic evaluation of ALS. It is crucial to identify the disease early and reliably, through metabolic patterns that allow us to confirm the disease, assess the coexistence of diseases that are frequently associated with ALS, such as FTD, and at the same time differentiate it from other pathologies with similar clinical characteristics.

## Data Availability

The raw data supporting the conclusions of this article will be made available by the authors, without undue reservation.

## Supplementary material

Supplementary Video 1: Maximum intensity projection (MIP) Brain 18F-FDG PET.
